# Introductory Lectures

**Published:** 1847-12

**Authors:** 


ARTICLE V.

### INTRODUCTORY LECTURES.

We have received introductory Lectures, delivered the present Session by Professors Lawson and Harrison, of the Medical College of Ohio, Professor Drake of the University of Louisville, and Professors Dunglison, Mitchell and Mütter, of the Jefferson Medical College of Philadelphia, all published by the class, indicating quite an increase in this branch of medical literature, which generally corresponds to its poetry, being designed to please as well as to instruct.

Professor Lawson's is full of poetry of thought and language, the evident emenation from an active energetic mind.

Professor Harrison's treats of the "state and prospects of the Medical profession." He sees in the signs of the times much to encourage, and refers to the moral as well as intellectual improvements that are being made.

He approves of the multiplication of Medical Schools, when in a proper location and properly organized, as affording many great and important advantages to the progress of the profession, and in repressing quackery. It is a good address and shows well the bright side of the picture of the profession.

Prof. Drake's is truly, as its title proclaims, "strictures on some of the defects and infirmities of intellectual and moral character in students of medicine," and is applied to his class in groups (leaving the members to arrange themselves according to the lines marked out,) of which there are eleven, which severally receive "their portion in due season."

Group 1st. is composed of the gifted, and the 2nd of third course students, who are advised to beware lest they relax their efforts in consequence of ability and advantages—the 3rd, practitioners—the 4th, of those who are without due preparation—the 5th, of those who are under the necessity of going to practice before completing their studies—the 6th, of those too intent on practical matters—the 7th, of the mentally incompetent—the 8th, of those who lack punctuality—the 9th, of those fond of social conviviality—the 10th, of the dissipated, and the 11th, of the bullies and desperadoes, who severally are shown up in that masterly manner peculiar to the author, and are encouraged, advised, admonished, reproved or *scathed*, as their several cases seem to demand ; and the address closes with a few reflections on the defects that are common to them all. It is sound, practical, strong and clear, such as we always have a right to expect, when we see Dr. Drake's name on the cover.

Professor Dunglison's is a learned history of the healing art, and correctly points out the use to be made by the man of science, of the 'isms, 'pathys and nostrums, by waiting until the mystery is removed, and appropriating to his own use, all the valuable that can be gleaned from amongst the dross. He commences by a few preliminary remarks, in which he refers to that "largest class" that had ever assembled in the United States.

Professor Mitchell after the preliminary reference to the prospects of the school and the "largest class that ever presented itself before a medical teacher on this side of the Atlantic," speaks at length in reference to, and seems to oppose, the lengthening of the terms of our college sessions, and winds up with quite a eulogy upon the profession in America. It is rather desultory, the author, Quaker fashion, not having taken a text to preach from ; showing however, that he is a clear thinker and a fluent writer.

Professor Mütter's, is devoted to the consideration of the qualifications necessary for the successful prosecution of the art. In the first place he shows the necessity for a good physical constitution, then of a thorough education, and shows the tendency of our science to expand the mind, citing that a large number of the most distinguished names on the scroll of fame, in almost all departments of intellectual greatness, are from the ranks of our profession. He shows the power of persevering industry in its strongest light, and closes with an exhortation to "strict morality and virtue" It is one of the best.

We are sorry our limits will not allow us to furnish a more extended notice of those pleasing and instructive evidences that our professional brethren are both actively laboring and highly appreciated. E.

